# Editorial: Crop improvement by omics and bioinformatics

**DOI:** 10.3389/fpls.2024.1391334

**Published:** 2024-04-03

**Authors:** Jun Li, Yan Zhao, Zhichao Wu, Xueqiang Wang

**Affiliations:** ^1^ Hainan Institute of Zhejiang University, Sanya, Hainan, China; ^2^ Zhejiang Provincial Key Laboratory of Crop Genetic Resources, the Advanced Seed Institute, Plant Precision Breeding Academy, College of Agriculture and Biotechnology, Zhejiang University, Hangzhou, Zhejiang, China; ^3^ State Key Laboratory of Crop Biology, Shandong Key Laboratory of Crop Biology, College of Agronomy, Shandong Agricultural University, Tai’an, Shandong, China; ^4^ National Cancer Institute, National Institutes of Health, Bethesda, MD, United States; ^5^ Yazhouwan National Laboratory, Sanya, Hainan, China

**Keywords:** omics, bioinformatics, population genetics, improvement, evolution

## 1 Introduction

Crop improvement in modern era by the genetic and breeding tools is being driven by the requirements of food security and sustainability. The caloric and nutritional needs of the growing population, and respond to environmental changes are the two demands of crop productivity (Zeng et al., 2017; Wang et al., 2018; Chen et al., 2021; Ren et al., 2023). In order to meet these demands, the global food production must increase by one billion tons in the next few decades, but the current growth rate is far from being reached. Moreover, rapid changes in the environment are accelerating land degradation, aggravating pests and diseases, introducing extreme stress and reducing crop productivity (Zeng et al., 2017; Zelm et al., 2020; Liang et al., 2021; Wang et al., 2023).

In the past few decades, remarkable progresses have been achieved in the discovery of yield, quality and resistance genes in crops, and the dissection of plant molecular mechanisms (Zeng et al., 2017; Wang et al., 2018; Zelm et al., 2020; Chen et al., 2021; Liang et al., 2021; Ren et al., 2023; Wang et al., 2023). With the continuous progress in sequencing technology, molecular markers and gene editing, a large number of excellent crop varieties have been cultivated and modern genetic improvement of crops have been realized (Lei et al., 2021; Qin et al., 2021; Tang et al., 2022; Wang and Han, 2022; Shi et al., 2023; Wen et al., 2023). But it is far from enough compared with the rapid changes in the growing population and environment.

Many new omics technologies have been developed in recent years, e.g., genomics, transcriptomics, proteomics, metabolomics, interactomics, and phenomics (Xie et al., 2021; Huang et al., 2022; Shang et al., 2022; Wang and Han, 2022; Wang et al., 2022; Marand et al., 2023; Ren et al., 2023). Integrating multi-omics could clarify the mechanisms of many biological processes and explore the interactions among various substances (Della Coletta et al., 2021; Huang et al., 2022; Luo et al., 2022). This will provide a new perspective for understanding the complex traits of crops and accelerate the breeding programs. The crop improvement is entering a new era of biological information (Shang et al., 2022; Wang and Han, 2022; Shi et al., 2023; Wen et al., 2023).

In this editorial, we set up a Research Topic of Crop Improvement by Omics and Bioinformatics. The goal of this Research Topic is to collect all types of research and review articles describing the latest advances in the discovery of yield, quality and resistance genes in crops, and the dissection of crop molecular mechanisms. In addition, recent discoveries derived from the development or application of new omics technologies in crops as well as new methods for the analysis, mining, and visualization of crop omics datasets are also delightedly accepted. The following themes are included in this Research Topic: (a) Population genetics, haplotype analysis and evolution of important genes in crops; (b) Development of new omics technologies (software or algorithm) for crop improvement; (c) Multi-omics approaches to understand the molecular basis of the genes for important agronomic traits in crops; (d) Integration with multi-omics revealing the origin and evolution of crops; (e) Meta-analysis and comparative analysis of crop omics datasets.

## 2 Discovery of important genes by multi-omics approaches

Chen et al. systematically evaluated various state-of-the-art object detectors on rice panicle counting and identified YOLOv8-X as the optimal detector. Applying YOLOv8-X to UAV time-series images of 294 rice recombinant inbred lines (RILs) allowed accurate quantification of six heading date-related traits and identified quantitative trait loci (QTL), including verified loci and novel loci, associated with heading date. This research optimized UAV phenotyping and computer vision pipeline that may facilitate scalable molecular identification of heading-date-related genes and guide enhancements in rice yield and adaptation.

Li et al. evaluated the heat tolerance at the seedling stage using 620 diverse rice accessions, and based on the GWAS and transcriptomics integrated results, a hypothetical model modulated by *qHT7* in response to heat stress was proposed. The results provided valuable candidate genes for improving rice heat tolerance through molecular breeding.

Yu et al. identified 5, 6, 6, and 6 QTLs for grain length, grain weight, grain area, and thousand grain weight, respectively, using 55K SNP assay genotyping and large scale phenotyping data of the population and GWAS. A comprehensive analysis of transcriptome data and homologs showed that *TraesCS2D02G414800* could be the real QTL gene for *qGL-2D*. Overall, this study presented several reliable grain size QTLs and candidate genes for grain length for future bread wheat breeding for yield.

Sun et al. screened a total of 15 candidate genes from a genome-wide association study (GWAS) on 8 traits of 150 cotton germplasms under drought conditions and found four genes were highly expressed after drought stress. Three of these genes had the same differential expression pattern. This study provides a theoretical basis for the genetic analysis of cotton yield traits under drought stress, and provides gene resources for improved breeding of cotton yield traits under drought stress.

Wu et al. identified 25 potential earliness related genes from Chinese bayberry (*Myrica rubra*) by analyzing the transcriptome data from early ripening, medium ripening and late ripening varieties, with clustering analysis and comparisons of genes reported related to flowering in *Arabidopsis thaliana*. Finally, through transgenic studies, this study identified an important gene *MrSPL4* in Chinese bayberry, which enhanced growth and flowering, providing important theoretical basis for early-mature breeding of Chinese bayberry.

Gao et al. conducted metabolomic and transcriptomic analyses of 5~8 years old *Cinnamomum cassia*, in order to explore the mechanism of the dynamic accumulation of active ingredients. A total of 72 phenylpropanoids, 146 flavonoids, and 130 terpenoids were found to exhibit marked changes. In addition, transcription factors (TFs) and genes involved in phenylpropanoids and flavonoids synthesis and regulation were identified through co-expression network analyses. The results of this study provide new insights into the synthesis and accumulation of phenylpropanoid, flavonoids and terpenoids in *C. cassia* at four growth stages.

Gao et al. performed full-length transcriptome analysis of *in vitro* bulblet initiation in lily. They compared the expression profiles of crucial genes of carbohydrate metabolism between different stages and different treatments. Significant co-expression was shown between genes involved in carbohydrate metabolism and auxin signaling, together with transcription factors such as bHLHs, MYBs, ERFs and C3Hs. This study indicates the coordinate regulation of bulblet initiation by carbohydrate metabolism and auxin signaling, serving as a basis for further studies on the molecular mechanism of bulblet initiation in lily and other bulbous flowers.

Li et al. presented a co-expression network, involving ABA and other phytohormone signals, based on weighted gene co-expression network analysis of spatiotemporally resolved transcriptome data and phenotypic changes of strawberry receptacles during development and following various treatments. They explored the role of two hub signals, small auxin up-regulated RNA 1 and 2 in receptacle ripening mediated by ABA, which are also predicted to contribute to fruit quality. These results and publicly accessible datasets provide a valuable resource to elucidate ripening and quality formation mediated by ABA and multiple other phytohormone signals in strawberry receptacle and serve as a model for other non-climacteric fruits.

## 3 Gene family analysis

Liu et al. identified a total of 18 *Whirly* genes from six Triticeae species and found TaWHY1-7A and TaWHY1-7D mainly enhanced the tolerance to oxidative stress in yeast cells. TaWHY2s mainly improved NaCl stress tolerance and were sensitive to oxidative stress in yeast cells. The heterologous expression of *TaWHY1-7D* greatly improved drought and salt tolerance in transgenic *Arabidopsis*. These results provide the foundation for further functional study of *Whirly* genes aiming at improving osmotic stress tolerance in wheat.

Liang et al. identified 37 *StSOS1s* in potato (*Solanum tuberosum*), which were found to be unevenly distributed across 10 chromosomes, with the majority located on the plasma membrane. RT-qPCR results revealed that the expression of *StSOS1s* were significant modulated by various abiotic stresses, in particular salt and abscisic acid stress. This work extends the comprehensive overview of the *StSOS1* gene family and sets the stage for further analysis of the function of genes in SOS and hormone signaling pathways.

Tang et al. identified 57 CCCH genes in the pepper (*Capsicum annuum* L.) genome and explored the evolution and function of the CCCH gene family in *C. annuum*. They found that the expression of CCCH genes was significantly up-regulated during the response to biotic and abiotic stresses, especially cold and heat stresses, indicating that CCCH genes play key roles in stress responses. These results provide new information on CCCH genes in pepper and will facilitate future studies of the evolution, inheritance, and function of CCCH zinc finger genes in pepper.

Zhang et al. identified 59 bZIP genes that were unevenly distributed in the chestnut genome, and found *CmbZIP04*, *CmbZIP13*, *CmbZIP14*, *CmbZIP33*, *CmbZIP35*, *CmbZIP38*, and *CmbZIP56* may be important in regulating starch accumulation in chestnut seeds. This study provided basic information on CmbZIP genes, which can be utilized in future functional analysis and breeding studies.

## 4 Development of the omics technologies

Shen et al. presented the application of alternative splicing algorithms with or without reference genomes in plants, as well as the integration of advanced deep learning techniques for improved detection accuracy, and discussed alternative splicing studies in the pan-genomic background and the usefulness of integrated strategies for fully profiling alternative splicing.

Zhang et al. induced male sterile mutants by simultaneously editing three cotton *EXCESS MICROSPOROCYTES1* (*GhEMS1*) genes by CRISPR/Cas9. This study would not only facilitates the exploration of the basic research of cotton male sterile lines, but also provides germplasms for accelerating the hybrid breeding in cotton.

Liu et al. developed a new genomic prediction method (RHEPCG) via combining randomized Haseman-Elston (HE) regression (RHE-reg) and preconditioned conjugate gradient (PCG), which avoids the direct inverse of the genomic relationship matrix (GRM). The simulation results demonstrated that RHEPCG not only achieved similar predictive accuracy with GBLUP in most cases, but also significantly reduced computational time, indicating that RHEPCG is a practical alternative to GBLUP with better computational efficiency.

Aparicio et al. developed the Mr.Bean, an accessible and user-friendly tool with a comprehensive graphical visualization interface, to predict the genetic potential of evaluated genotypes. The application integrates descriptive analysis, measures of dispersion and centralization, linear mixed model fitting, multi-environment trial analysis, factor analytic models, and genomic analysis, aiming at helping plant scientists working in agricultural field make informed decisions more quickly.

## 5 Perspective

It is crucial to identify yield, quality and resistance related genes in crops, and dissect the involved molecular mechanisms. In addition, recent discoveries derived from the development or application of new omics technologies in crops as well as new methods for the analysis, mining, and visualization of crop omics datasets are also urgently needed. These results will provide a new perspective for understanding the complex traits of crops and accelerate the breeding programs.

## Author contributions

XW: Conceptualization, Formal analysis, Funding acquisition, Investigation, Methodology, Supervision, Validation, Visualization, Writing – review & editing. JL: Conceptualization, Formal analysis, Investigation, Methodology, Writing – original draft. YZ: Conceptualization, Data curation, Formal analysis, Investigation, Writing – original draft. ZW: Conceptualization, Investigation, Methodology, Resources, Writing – original draft.
